# Prevalence of chronic obstructive pulmonary disease and variation in risk factors across four geographically diverse resource-limited settings in Peru

**DOI:** 10.1186/s12931-015-0198-2

**Published:** 2015-03-18

**Authors:** Devan Jaganath, J Jaime Miranda, Robert H Gilman, Robert A Wise, Gregory B Diette, Catherine H Miele, Antonio Bernabe-Ortiz, William Checkley, CRONICAS Cohort Study Group

**Affiliations:** Division of Pulmonary and Critical Care, School of Medicine, Johns Hopkins University, 1800 Orleans Ave Suite 9121, Baltimore, MD 21205 USA; Program in Global Disease Epidemiology and Control, Department of International Health, Bloomberg School of Public Health, Johns Hopkins University, Baltimore, USA; CRONICAS Center of Excellence for Chronic Diseases, Universidad Peruana Cayetano Heredia, Lima, Peru; Departamento de Medicina, Escuela de Medicina, Universidad Peruana Cayetano Heredia, Lima, Peru

**Keywords:** COPD, Peru, Respiratory disease, Chronic disease, Risk factors, Population studies, Biomass fuels

## Abstract

**Background:**

It is unclear how geographic and social diversity affects the prevalence of chronic obstructive pulmonary disease (COPD). We sought to characterize the prevalence of COPD and identify risk factors across four settings in Peru with varying degrees of urbanization, altitude, and biomass fuel use.

**Methods:**

We collected sociodemographics, clinical history, and post-bronchodilator spirometry in a randomly selected, age-, sex- and site-stratified, population-based sample of 2,957 adults aged ≥35 years (median age was 54.8 years and 49.3% were men) from four resource-poor settings: Lima, Tumbes, urban and rural Puno. We defined COPD as a post-bronchodilator FEV_1_/FVC < 70%.

**Results:**

Overall prevalence of COPD was 6.0% (95% CI 5.1%–6.8%) but with marked variation across sites: 3.6% in semi-urban Tumbes, 6.1% in urban Puno, 6.2% in Lima, and 9.9% in rural Puno (p < 0.001). Population attributable risks (PARs) of COPD due to smoking ≥10 pack-years were less than 10% for all sites, consistent with a low prevalence of daily smoking (3.3%). Rather, we found that PARs of COPD varied by setting. In Lima, for example, the highest PARs were attributed to post-treatment tuberculosis (16% and 22% for men and women, respectively). In rural Puno, daily biomass fuel for cooking among women was associated with COPD (prevalence ratio 2.22, 95% CI 1.02–4.81) and the PAR of COPD due to daily exposure to biomass fuel smoke was 55%.

**Conclusions:**

The burden of COPD in Peru was not uniform and, unlike other settings, was not predominantly explained by tobacco smoking. This study emphasizes the role of biomass fuel use, and highlights pulmonary tuberculosis as an often neglected risk factor in endemic areas.

## Introduction

The chronic disease epidemic has placed a large burden on morbidity and mortality in low and middle-income countries. The 2010 Global Burden of Disease study indicated that 54% of all disability-adjusted life years lost worldwide were attributed to non-communicable, chronic diseases [1]. Chronic obstructive pulmonary disease (COPD), characterized by lower airway inflammation and damage that impairs airflow, is the third leading cause of death worldwide, with a global prevalence estimate of 10% [2,3]. Risk factors for COPD reported in the literature include smoking, outdoor pollution, indoor pollution, occupational dust exposure, asthma, past history of tuberculosis, and genetics [4-6].

Low- and middle-income countries currently share the greatest proportion of chronic respiratory disease worldwide [7,8]. Management guidelines and public health efforts specific to these settings are critical to prevention and care for COPD [9]. However, healthcare infrastructure for COPD surveillance and management in resource-limited settings are lacking [7]. In addition, the PLATINO and BOLD studies demonstrated substantial geographic variation in COPD prevalence, and the PURE study similarly found that lung function differed greatly by region [6,10] This heterogeneity underscores the importance of investigating local factors that shape the burden of COPD, especially in resource-limited settings that contain considerable diversity in culture, socioeconomic status, and environment.

In Latin America, the PLATINO and PREPOCOL studies assessed the burden of COPD and highlighted risk factors such as tobacco smoking across their study settings [11-13]. However, there is a critical gap in our understanding of COPD across diverse geographical and social settings. Peruvians live in densely populated urban areas as well as rural village settings, along the coast and in the Andes Mountains, and we sought to characterize variation in prevalence and risk factors for COPD.

## Methods

### Setting

The CRONICAS study is a longitudinal, population-based study aimed to determine the prevalence of chronic pulmonary and cardiovascular diseases across four disparate regions in Peru. The study has been described in detail elsewhere [14] and involved collection of sociodemographic and clinical data from adults aged ≥35 years in four diverse populations in Peru. The four settings under study varied based on degrees of urbanization, living at high altitude, prevalence of daily biomass fuel use. Pampas de San Juan de Miraflores is a dense, urbanized community 25 kilometers south of central Lima and consists primarily of Andean immigrants. Tumbes is a semi-urban, sea-level community in northern Peru within an agrarian setting and little to no vehicular traffic. Puno is a southwestern city in the Andes, located on the shores of Lake Titicaca at 3,825 meters above sea-level. Within Puno there were two separate sites: an urban setting located at the city center and a rural setting made up of inhabitants from surrounding communities [15].

### Design

We enrolled an age (35–44, 45–54, 55–64 and ≥65 years), sex and site stratified sample of eligible subjects. Recruitment began in September 2010 and was conducted until about 1,000 participants with complete information per site were enrolled. Only one participant per household was enrolled. In Puno, we stratified recruitment to include 500 participants each from the urban and rural settings. Inclusion criteria were age ≥35 years, a full-time resident in the specified setting, and capacity to understand procedures and consent to the study. Exclusion criteria were pregnancy, physical disability that prevented measurement of blood pressure or anthropometry, or active pulmonary tuberculosis. All participants provided verbal informed consent after our research team read the entire informed consent document to them and any questions were answered. Informed consents were verbal because of high illiteracy rates. The study was approved by the Institutional Review Boards of Universidad Peruana Cayetano Heredia and A.B. PRISMA, in Lima, Peru, and the Johns Hopkins Bloomberg School of Public Health in Baltimore, USA.

### Data collection

Participants responded to a questionnaire on sociodemographics, smoking history, respiratory symptoms, past medical history, and family history of disease. A detailed questionnaire also evaluated frequency and duration of cooking with biomass fuels. Field workers measured weight and height in triplicate. Spirometry was conducted using the Easy-On-PC spirometer (ndd, Zurich, Switzerland) before and after 200 mcg of inhaled salbutamol via a spacer. Trained technicians measured pre- and post-bronchodilator spirometry in participants following joint American Thoracic Society and European Respiratory Society (ATS/ERS) guidelines [16]. We adapted a standardized grading system for quality control, review and interpretation [17]. Participants with low quality spirometry were asked to repeat the test on another day for a total of three attempts. Overall, 96% of all tests achieved an acceptable result and 95% met ATS/ERS criteria.

### Definitions

We defined COPD according to the Global initiative for chronic Obstructive Lung Disease (GOLD) criteria as a post-bronchodilator FEV_1_/FVC < 70% [18]. We used percent-predicted post-bronchodilator FEV_1_ values to assess severity by GOLD staging. Since there are no established reference equations for Peruvians, we utilized both the NHANES III Mexican-Americans [19] and the Global Lungs Initiative (GLI) mixed ethnic population equations [20]. In a sensitivity analysis, we also defined COPD as post-bronchodilator FEV_1_/FVC less than the lower limit of normal by age and sex according to GLI mixed ethnic population equations [20]. We defined a post-bronchodilator response as an improvement of greater than 12% and 200 mL in post-bronchodilator vs. pre-bronchodilator forced expiratory volumes. We defined high altitude as either living in urban or rural Puno versus sea level as either living in Lima or Tumbes. We derived a wealth index based on assets, household facilities and household income [21].

### Biostatistical methods

The primary objectives of this analysis were to estimate the prevalence of COPD and identify variation in risk factors across our settings. We estimated the prevalence of COPD and corresponding 95% confidence intervals (CI) by site using standard methods. We compared baseline characteristics and spirometry results across sites using chi-squared or Fisher exact tests for categorical variables and Wilcoxon rank sum or Kruskal-Wallis tests for continuous variables. We used sex-stratified multivariable Poisson regressions [22] to model the prevalence of COPD as a function of age, living at high altitude, degree of urbanization, smoking for at least 10 pack-years, tertiles of wealth index, history of post-treatment pulmonary tuberculosis, history of asthma, and daily use of biomass fuels for cooking. We used the Huber-White method to correct the variance-covariance matrix for correlated responses by site [23]. We did not include indicator variables for site as fixed effects in the multivariable regression model because of collinearity with selected environmental risk factors (Table 1). Population attributable risks for COPD due to risk factors were calculated as $PAR=\frac{p\left(PR-1\right)}{1+p\left(PR-1\right)},$ where p was the prevalence of exposure and PR was the adjusted prevalence ratio obtained from multivariable Poisson regression. Twenty-seven participants with missing values (<1%) of the 2,957 were excluded from multivariable analysis. In an ancillary analysis, we assessed inter-test reliability for severity staging when using NHANES III vs. GLI percent predicted values both by percent agreement and the kappa statistic. Analyses were performed in STATA 12 (StataCorp, College Station, Texas, USA) and R (www.r-project.org).

**Table 1:** **Selected environmental risk factors by setting**

**Setting**	**Urbanization**	**Daily use of biomass fuels**	**Altitude**
Lima	Urban	Rare	Sea level
Tumbes	Semi-urban	Moderately prevalent	Sea level
Puno, urban	Urban	Rare	3,825 meters above sea level
Puno, rural	Rural	Highly prevalent	3,825 meters above sea level

## Results

### Participant characteristics

**Figure 1:**
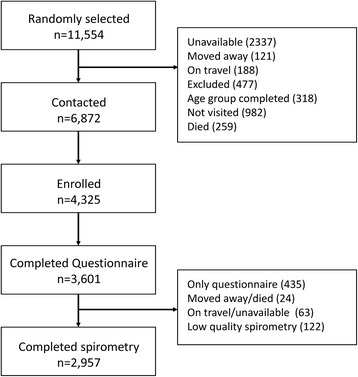
**Flowchart of participants.**

We enrolled 4,325 participants of whom 3,601 (83%) had complete questionnaires (Figure 1). In total, 2,957 participants had complete spirometry data. We did not identify differences in age (p = 0.72), sex (p = 0.15), daily smoking (p = 0.80), self-reported history of post-treatment pulmonary tuberculosis (p = 0.88) and asthma (p = 0.25) or biomass fuel use (p = 0.22) between participants with and without spirometry data in an analysis adjusted for site.

Among the 2,957 participants, median age was 54.8 years and 49.3% were men (Table 2). Daily smoking was low at 3.3% across all sites, and ranged from 0.2% in rural Puno to 5.6% in Tumbes (p < 0.001). Median pack-years among smokers was 0.2 (IQR 0.01–1.53). When stratified by sex, we found that 79 (5.5%) men reported smoking daily compared to only 18 (1.2%) women (p < 0.001). Overall, 2.9% of participants reported a history of post-treatment pulmonary tuberculosis and 3.9% reported a history of asthma. Participants in Lima reported having the highest prevalences of post-treatment pulmonary tuberculosis and asthma. Eight (0.4%) individuals reported having a family history of COPD.

**Table 2:** **Participant characteristics across four sites in Peru (p-values calculated using Kruskal-Wallis tests for continuous variables, and chi-squared or Fisher exact tests for categorical variables)**

	**Lima**	**Urban Puno**	**Rural Puno**	**Tumbes**	**Overall**	**P**
	**998 (33.8)**	**507 (17.2)**	**506 (17.1)**	**946 (32.0)**	**2957**	
**Age in years, median (IQR)**	54.7 (45.5, 64.0)	54.8 (44.9, 64.5)	54.8 (45.1, 63.9)	54.9 (45.0, 64.8)	54.8 (45.2, 64.3)	0.87
**Number of males (%)**	492 (49.3)	250 (49.3)	240 (47.4)	475 (50.2)	1457 (49.3)	0.80
**Height in m, median (IQR)**	1.54. (1.49, 1.61)	1.57 (1.50, 1.63)	1.55 (1.49, 1.62)	1.58 (1.52, 1.65)	1.56 (1.50, 1.63)	<0.001
**BMI >30 kg/m** ^**2**^ **, n (%)**	324 (32.5)	133 (26.4)	54 (10.8)	299 (31.6)	810 (27.5)	<0.001
**Daily smoking, n (%)**	32 (3.2)	11 (2.2)	1 (0.2)	53 (5.6)	97 (3.3)	<0.001
**Rural origin, n (%)**	510 (51.2)	103 (20.5)	481 (96.4)	285 (30.2)	1379 (46.9)	<0.001
**Wealth index, n (%)**						
Low	119 (11.9)	120 (23.9)	356 (70.4)	307 (32.5)	902 (30.6)	<0.001
Middle	366 (36.7)	129 (25.7)	136 (26.9)	387 (40.9)	1018 (34.5)	
High	513 (51.4)	254 (50.5)	14 (2.8)	252 (26.6)	1033 (35.0)	
**Exposures, n (%)**						
Daily use of biomass fuels for cooking	61 (6.1)	25 (5.0)	483 (96.6)	221 (23.4)	790 (26.8)	<0.001
Cooks indoors	573 (57.8)	436 (86.7)	453 (90.8)	497 (53.0)	1959 (66.8)	<0.001
**Clinical, (%)**						
History of tuberculosis	69 (6.9)	3 (0.6)	7 (1.4)	7 (0.7)	86 (2.9)	<0.001
History of asthma	82 (8.2)	13 (2.6)	3 (0.6)	16 (1.7)	114 (3.9)	<0.001
Family History of asthma	146 (24.7)	18 (3.6)	8 (1.6)	51 (7.9)	223 (10.0)	<0.001
Family History of COPD	4 (0.7)	4 (0.8)	0	0	8 (0.4)	0.04

Non-urban settings had the highest proportions of biomass fuel use: ≥97% of participants in rural Puno reported that biomass fuels were used daily for cooking in their household, and 91% reported cooking indoors. A total of 23% reported daily biomass fuel use in Tumbes, but 53% reported cooking indoors. Women were more likely to be the household cook: 1,432 (95.7%) reported that they cooked vs. 862 (59.4%) males (p < 0.001).

### Variation in lung function measurements

We compared post-bronchodilator lung function across sites in both men (Table 3) and women (Table 4). Living at high altitude (urban or rural Puno) vs. at sea level (Lima or Tumbes) was associated with having higher average FEV_1_ values in both males (3.5 L vs. 3.3 L; p < 0.001) and females (2.4 L vs. 2.3 L; p < 0.001), higher average FVC values in both males (4.4 L vs. 4.1 L; p < 0.001) and females (3.0 L vs. 2.7 L; p < 0.001), but lower average FEV_1_/FVC values in both males (78.0% vs. 80.2%; p < 0.001) and females (79.9% vs. 82.8%; p < 0.001).

**Table 3:** **Median and interquartile range of post-bronchodilator lung function values in men using the Global Lungs Function Initiative (GLI) mixed ethnic population and Hankinson NHANES III Mexican-American reference equations (p-values calculated using Kruskal-Wallis tests)**

	**Lima (n = 492)**	**Urban Puno (n = 250)**	**Rural Puno (n = 240)**	**Tumbes (n = 475)**	**P**
**FEV**_**1**_ **(L)**	3.4 (2.9–3.7)	3.5 (3.0–4.0)	3.5 (2.9–3.9)	3.2 (2.8–3.6)	<0.001
**FVC (L)**	4.2 (3.7–4.7)	4.4 (3.8–5.1)	4.4 (3.9–5.0)	4.0 (3.4–4.4)	<0.001
**FEV**_**1**_ **/height** ^**2**^ **(L/m** ^**2**^ **)**	1.3 (1.1–1.4)	1.3 (1.1–1.4)	1.3 (1.1–1.5)	1.2 (1.0–1.3)	<0.001
**FVC/height** ^**2**^ **(L/m** ^**2**^ **)**	1.6 (1.4–1.8)	1.7 (1.5–1.9)	1.7 (1.5–1.9)	1.5 (1.3–1.6)	<0.001
**FEV**_**1**_ **/FVC (%)**	80.0 (75.4–83.4)	78.4 (74.9–82.3)	77.8 (73.6–81.0)	80.6 (77.4–84.0)	<0.001
**Percent Predicted FEV**_**1**_ **(%)**					
GLI	117.9 (107.5–129.8)	119.9 (109.9–130.2)	121.7 (111.5–133.9)	107.7 (97.7–117.5)	<0.001
NHANES III	113.3 (102.0–124.7)	114.6 (105.2–124.2)	117.0 (106.7–129.0)	102.4 (92.6–112.0)	<0.001
**Percent Predicted FVC (%)**					
GLI	120.1 (108.9–130.5)	123.0 (112.8–134.3)	126.2 (114.4–139.3)	107.7 (98.3–116.0)	<0.001
NHANES III	109.7 (99.8–119.5)	112.3 (104.7–123.3)	116.1 (105.6–128.1)	98.7 (90.1–107.0)	<0.001

**Table 4:** **Median and interquartile range of post-bronchodilator lung function values in women using the Global Lungs Function Initiative (GLI) mixed ethnic population and Hankinson NHANES III Mexican-American reference equations (p-values calculated using Kruskal-Wallis tests)**

	**Lima (n = 506)**	**Urban Puno (n = 257)**	**Rural Puno (n = 266)**	**Tumbes (n = 471)**	**P**
**FEV**_**1**_ **(L)**	2.3 (2.0–2.6)	2.4 (2.0–2.8)	2.4 (2.0–2.8)	2.2 (1.9–2.5)	<0.001
**FVC (L)**	2.8 (2.4–3.2)	3.0 (2.5–3.5)	3.0 (2.5–3.5)	2.6 (2.3–2.9)	<0.001
**FEV**_**1**_ **/height** ^**2**^ **(L/m** ^**2**^ **)**	1.0 (0.9–1.2)	1.1 (0.9–1.2)	1.1 (0.9–1.2)	0.9 (0.8–1.1)	<0.001
**FVC/height** ^**2**^ **(L/m** ^**2**^ **)**	1.3 (1.1–1.4)	1.3 (1.1–1.5)	1.3 (1.2–1.5)	1.1 (1.0–1.3)	<0.001
**FEV**_**1**_ **/FVC (%)**	82.7 (79.3–85.3)	80.5 (77.0–83.6)	79.6 (75.6–82.5)	83.0 (79.7–86.1)	<0.001
**Percent predicted FEV**_**1**_ **(%)**					
GLI	112.0 (102.1–123.7)	115.2 (106.1–126.5)	113.9 (102.3–126.0)	103.1 (93.8–112.0)	<0.001
NHANES III	106.2 (96.9–117.2)	110.4 (100.7–119.7)	107.5 (96.5–118.4)	96.5 (88.5–105.5)	<0.001
**Percent predicted FVC (%)**					
GLI	111.7 (100.3–122.1)	119.2 (106.4–129.2)	116.9 (104.7–128.3)	101.1 (93.2–109.9)	<0.001
NHANES III	103.4 (92.8–113.0)	110.5 (98.3–119.1)	107.6 (96.7–118.4)	93.1 (85.5–100.8)	<0.001

### Variation in COPD prevalence

The overall prevalence of COPD was 6% with marked variation by sex and across sites. When stratified by sex, we found that 123 (8.4%) men and 54 (3.6%) women had COPD (p < 0.001). Across sites, prevalence ranged from 3.6% in Tumbes to 9.9% in rural Puno (Table 5). Participants in urban Puno had a similar prevalence of COPD than did those in Lima (p = 1.00) but a lower prevalence than participants in rural Puno (p = 0.04). This trend was similar for both men and women. Staging by GOLD criteria showed that the majority of cases were either Stage I or II and similar across sites, regardless of GLI mixed ethnic population or NHANES III Mexican American reference equations (93.8% agreement, kappa = 0.85). The only five severe stage cases of COPD (i.e., GOLD stage III) occurred in Lima and Tumbes, and all were male. We also found that post-bronchodilator response (i.e., reversibility) did not differ for males and females across sites among those with COPD. However, there was significant variation across sites for individuals without COPD and rural Puno had the highest proportion of participants with a post-bronchodilator response.

**Table 5:** **Prevalence of COPD across four sites in Peru using the Global Lungs Function Initiative (GLI) mixed ethnic population and Hankinson NHANES III Mexican-American reference equations (p-values calculated using chi-squared or Fisher exact tests)**

	**Lima**	**Urban Puno**	**Rural Puno**	**Tumbes**	**Overall**	**P**
	**(n = 998)**	**(n = 507)**	**(n = 506)**	**(n = 946)**		
**COPD prevalence (95% CI)**	6.2 (4.7–7.7)	6.1 (4.0–8.2)	9.9 (7.3–12.5)	3.6 (2.4–4.8)	6.0 (5.1–6.8)	<0.001
**GOLD class by GLI, n (%)**						
**I**	43 (69.4)	26 (83.9)	38 (76.0)	21 (61.7)	128 (72.3)	0.29
**II**	16 (25.8)	5 (16.1)	12 (24.0)	11 (32.4)	44 (24.9)	
**III**	3 (4.8)	0	0	2 (5.9)	5 (2.8)	
**GOLD class by NHANES III, n (%)**						
**I**	42 (67.7)	24 (77.4)	38 (76.0)	19 (55.9)	123 (69.5)	0.26
**II**	17 (27.4)	7 (22.6)	12 (24.0)	13 (38.2)	49 (27.8)	
**III**	3 (4.8)	0	0	2 (5.9)	5 (2.8)	
**Post-bronchodilator response in COPD, n (%)**	22 (36%)	8 (26%)	13 (26%)	9 (27%)	52 (29%)	0.63
**Post-bronchodilator response in non-COPD, n (%)**	99 (11%)	57 (12%)	78 (17%)	73 (8%)	307 (11%)	<0.001

When using the lower limit of normal for FEV_1_/FVC based on GLI reference equations, we found that there were 142 cases of COPD (prevalence of 4.8%; 95% CI 4.0 to 5.6). By site, the prevalence was 57 (5.7%) in Lima, 25 (4.9%) in urban Puno, 43 (8.5%) in rural Puno, and 17 (1.8%) in Tumbes. Overall, this corresponded to 97% agreement with the fixed ratio definition, and a kappa of 0.69.

### Risk factors associated with COPD

Single variable analysis suggested that age, sex, altitude, smoking, urbanization, socioeconomic status, post-treatment tuberculosis, asthma, and daily biomass fuel use may be associated with COPD prevalence (Table 6). In sex-stratified multivariable analyses (Table 7), older vs. younger individuals and those who lived at a higher altitude vs. at sea level had greater prevalence of COPD. Moreover, both a history of post-treatment tuberculosis and asthma were positively associated with COPD. Smoking for at least 10 pack-years was associated with COPD in males, and was marginally significant in females. We found that females but not males who lived in a rural setting were more likely to have COPD compared to an urban setting, while living in semi-urban region was associated with a decreased likelihood of COPD compared to an urban setting for both sexes.

**Table 6:** **Single variable factors associated with COPD prevalence across four sites in Peru (n = 2957)**

	**Prevalence ratio**	**95% CI**	**P**
**Age (per 10 years)**	1.93	1.71–2.17	<0.001
**Male vs. Female Sex**	2.35	1.61–3.42	<0.001
**Height (cm)**	1.01	0.99–1.02	0.27
**High Altitude vs. sea-level**	1.61	0.92–2.82	0.10
**≥10 pack-years of smoking**	2.51	1.65–3.80	<0.001
**Urbanization**			
Urban	1.00		
Semi-Urban	0.58	0.57–0.58	<0.001
Rural	1.57	1.56–1.59	<0.001
**Post-treatment pulmonary tuberculosis**	4.27	3.09–5.88	<0.001
**Asthma**	2.49	1.59–3.88	<0.001
**Biomass use at least once daily**	1.68	1.25–2.25	0.001
**Wealth Index**			
Low	1.00		
Middle	0.81	0.50–1.30	0.38
High	0.53	0.33–0.87	0.01

**Table 7:** **Multivariable regression of risk factors associated with COPD prevalence in Peru, stratified by sex (n = 2930)**

	**Males (n = 1443)**			**Females (n = 1487)**		
	**Prevalence ratio**	**95% CI**	**P**	**Prevalence ratio**	**95% CI**	**P**
**Age (per 10 years)**	1.65	1.42–1.92	<0.001	2.51	1.86–3.38	<0.001
**High altitude vs. sea-level**	1.13	1.06–1.21	<0.001	1.44	1.05–1.99	0.02
**≥10 pack-years of smoking**	1.80	1.40–2.32	<0.001	2.39	0.59–9.63	0.22
**Urbanization**						
Urban	1.00			1.00		
Semi-Urban	0.52	0.43–0.64	<0.001	0.66	0.53–0.82	<0.001
Rural	1.01	0.74–1.37	0.97	1.71	1.47–1.99	<0.001
**Post-treatment pulmonary tuberculosis**	3.12	2.02–4.83	<0.001	7.02	3.63–13.59	<0.001
**Asthma**	2.16	1.11–4.23	0.02	2.30	1.12–4.72	0.02
**Biomass use at least once daily**	1.08	0.55–2.11	0.82	2.22	1.02–4.81	0.04
**Wealth index**						
Low	1.00			1.00		
Middle	1.02	0.69–1.52	0.91	1.78	0.52–6.00	0.36
High	0.68	0.39–1.19	0.18	1.44	0.54–3.86	0.47

### Population attributable risks of COPD

**Figure 2:**
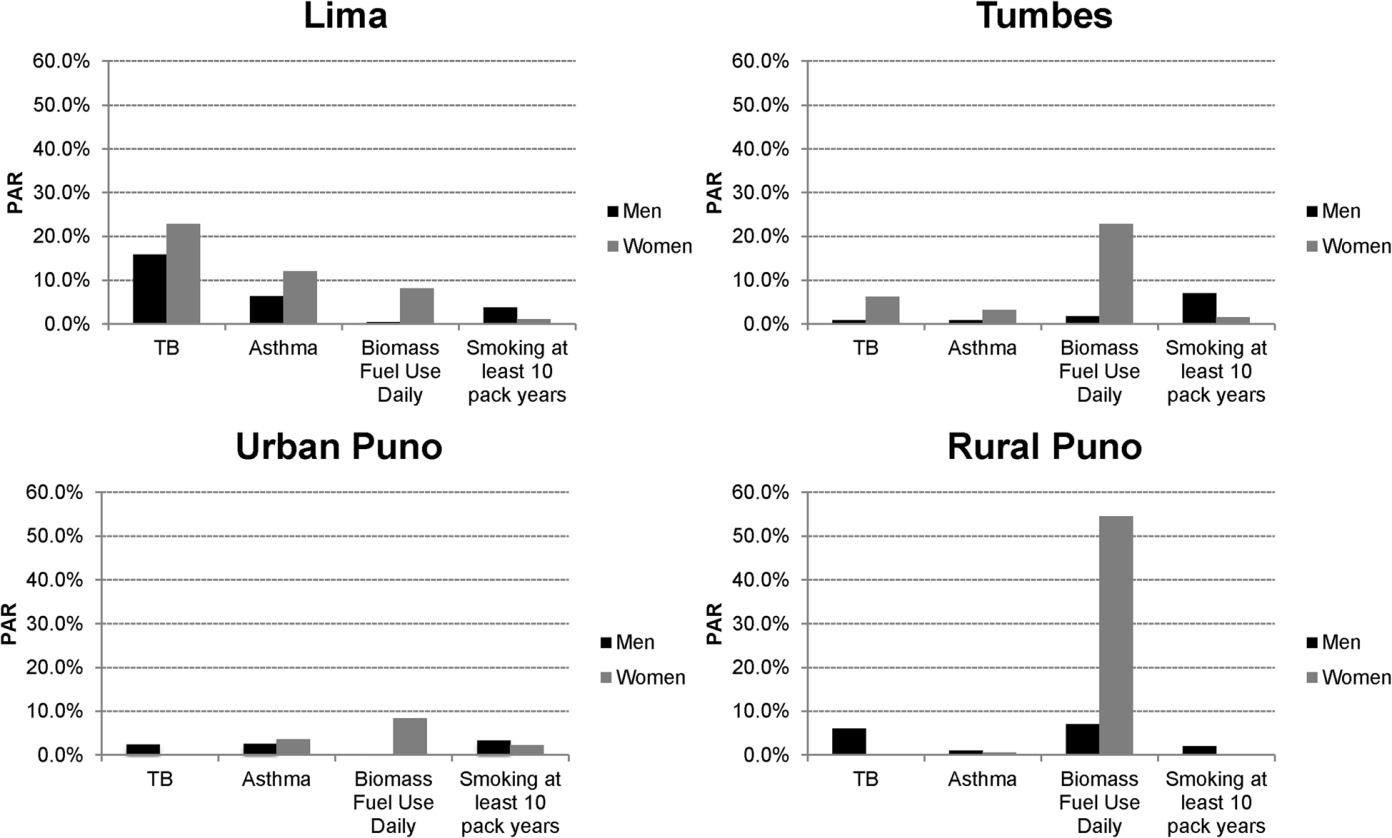
**Population attributable risk (PAR) among factors associated with COPD in Peru by setting and sex.**

We calculated PARs of COPD across sites and by sex due to daily biomass fuel use, smoking at least 10 pack-years, post-treatment pulmonary tuberculosis and asthma (Figure 2). Daily biomass fuel use notably increased PAR of COPD among women based on degree of urbanization, with 23% in semi-urban Tumbes and 55% in rural Puno. PAR of COPD due to smoking ≥10 pack years was less than 10% for all sites, and was greatest for men in Tumbes at 7%. Post-treatment pulmonary tuberculosis contributed the most to COPD in Lima, where PAR of COPD was 16% in men and 23% in women. PAR of COPD due to asthma was similarly highest in Lima, with 6% in men and 12% in women.

## Discussion

In this multi-center, population-based study of adult participants in Peru, we found considerable variation in lung function and prevalence of COPD among four resource-poor sites with varying degrees of urbanization, altitude, and biomass fuel use. While daily smoking prevalence was low across sites, population attributable risks of COPD suggest that daily biomass fuel use contributed to an important proportion of COPD in rural settings, whereas a history of post-treatment pulmonary tuberculosis and asthma were important risk factors in urban settings such as Lima. Our study also highlights the importance of post-treatment pulmonary tuberculosis, a prevalent disease with worldwide distribution, as a possible neglected risk factor for COPD in endemic areas.

The prevalence of COPD in Peru was 25% to 70% lower relative to multi-center studies in other countries [3,11]. In the BOLD study, the prevalence of COPD was 10.1% for moderate to severe (i.e., GOLD stage II or greater) in twelve sites from North America, Europe, Asia and Africa [3]. In the PLATINO study, the prevalence of COPD ranged from 7.8% to 19.7% in five cities from Brazil, Chile, Mexico, Uruguay and Venezuela [11]. In the PREPOCOL study, the prevalence of COPD was 8.9% across five Colombian cities at varying altitudes [13]. A possible reason for the low prevalence of COPD in our study is also the low prevalence of daily tobacco smoking: 3% were daily smokers, with a population attributable risk of COPD due to tobacco smoking of <10% across all sites for both men and women. In comparison, every city in the PLATINO study had a current smoking prevalence of >20% [24]. If smoking prevalence is low in Peru, this raised the question of what other determinants are associated with COPD, and what factors explained heterogeneity between sites. Utilizing within-country diversity, we evaluated this question to direct prevention and management efforts across settings.

Between our sites, we also found considerable variation in lung function and COPD prevalence. High altitude areas of urban and rural Puno had notably larger lung capacity as compared to sea level Lima or Tumbes, and supports past studies on physiological adaptation to high altitude [25,26]. Despite greater lung capacity and the lowest rates of smoking, FEV_1_/FVC was lower at higher altitude vs. sea level settings. The role of high altitude on COPD has been equivocal: investigators of the PLATINO study suggested that high altitude contributed to lower prevalence of COPD, whereas those of the PREPOCOL study found a higher prevalence of COPD at the high altitude sites of Colombia [11,13]. In both studies, the authors noted that changes in FEV_1_ and FVC among people who live at high altitude may affect classification of obstruction. We also found that high attitude vs. sea level was associated with a small but relatively greater increase in FVC compared to FEV_1_, which may lead to overestimation of COPD. At the same time, in the PLATINO and PREPOCOL studies, the highest altitude cities were Mexico City (2,250 meters above sea level) and Bogotá (2,640 meters above sea level), respectively, which are more urbanized than our high altitude setting in Puno (3,825 meters above sea level). Further research is required to investigate how altitude affects variation in COPD prevalence.

In our analysis, we also found that rural residents in Puno primarily cooked indoors and used biomass fuel daily. Notably, they had a higher prevalence of COPD than participants in urban Lima or even urban Puno. Biomass fuel is associated with a chronic inflammatory response and ultimately pulmonary damage that increase the risk of COPD [27,28]. In addition to biomass fuel smoke exposure, multivariable analysis revealed that a history of post-treatment pulmonary tuberculosis and asthma were also associated with COPD, specifically in our urban settings. Population attributable risks of COPD between sites further demonstrated the importance of post-treatment pulmonary tuberculosis and asthma in the urban setting of Lima. Pulmonary tuberculosis is associated with lung scarring, bronchial stenosis and parenchymal damage that can promote airway obstruction in endemic areas [4,29]. In the BOLD, PLATINO and PREPOCOL studies, investigators also found a positive association between pulmonary tuberculosis and COPD [5,13,29]. Chronic asthma has been associated with irreversible airway obstruction over time, with more rapid decline in FEV_1_ [4]. For both post-treatment pulmonary tuberculosis and asthma, further work will need to identify how their pathogenesis of airway obstruction compares to other traditional risk factors for COPD such as tobacco smoking [4], while well-designed prospective studies are necessary to assess direction of causality.

We also found sex-specific risk factors for COPD, which suggests that men and women in our setting face different types and amounts of exposures that impact their likelihood of disease. Among men, increasing pack years of smoking was an important determinant of COPD. While smoking among women was also associated with COPD, the effect was not significant, and may be a consequence of the low number of women (<2%) who smoked daily. The population attributable risk of COPD due to tobacco smoking was greatest in Tumbes, in particular among men where the local prevalence of smoking at least 10 pack-years was 10%. Among women, biomass fuel use at least once daily was also associated with COPD. Men did not show this trend, and suggests that while all members of the household were exposed to biomass fuel smoke, men may have an overall reduced exposure as they are commonly not the primary cook. A systematic review found that rural women who used biomass fuels had greater odds of having COPD [30]. Similarly, we found the population attributable risk of COPD due to daily exposure to biomass fuel smoke was 55% among women in rural Puno.

Our study has several strengths. First, our study is a population-based sample derived from four diverse geographical and social settings across Peru. Second, we collected extensive demographic, clinical, and behavioral data while conducting standardized pulmonary function tests to characterize COPD. Third, the low prevalence of tobacco smoking provided a unique opportunity for us to examine other risk factors associated with the prevalence of COPD. Our study also has some limitations. First, we were not powered to determine risk factors stratified by site. Second, we utilized two commonly used international reference equations to calculate the percent-predicted FEV_1_ and FVC [19,20]; however, the construction of these reference equations did not include Peruvians, which affect our estimation of severity categories. However, we found overall good consistency in the distributions of severity categories between the two reference equations. Third, self-reported history of COPD, post-treatment pulmonary tuberculosis or asthma has limitations compared to direct diagnostic evaluation and may be underestimated in those without access to care. Fourth, while sites varied in degree of urbanization and exposure to biomass fuel smoke and provided a gradient in ambient air pollution, personal air pollution exposure measurements would have provided a more quantitative comparison of differences. Finally, as a cross-sectional analysis, there are limitations in determining direction of causality for possible risk factors.

## Conclusion

We found that both the prevalence of COPD and risk factors in Peru varied significantly across settings. Daily biomass fuel smoke exposure, particularly in rural sites, was an important cause for this heterogeneity, as compared to differences in tobacco smoking. At the same time, a history of respiratory disease including post-treatment pulmonary tuberculosis and asthma were important factors in urban centers. Overall, we have highlighted unique aspects of COPD across geographic settings in Peru, to better understand these determinants, guide future studies, and implement interventions to reduce the burden of COPD.

## Abbreviations

COPD: Chronic obstructive pulmonary disease
FEV1: Forced expiratory volume in 1 second
FVC: Forced vital capacity
FEV_1_/FVC: Ratio of forced expiratory volume in 1 second to forced vital capacity
GOLD: Global initiative for chronic obstructive lung disease
GLI: Global Lungs Function Initiative
NHANES: National Health and Nutrition Examination Survey
CI: Confidence interval
